# Foreign

**Published:** 1841-09-04

**Authors:** 


### FOREIGN.

### Dr. Watson on Hæmorrhage.

You are to observe that I propose to treat of *hæmorrhage*, only so far as it falls to the care of the *physician*. The subject is exceedingly full of interest in its relations to surgery; and it will receive at the hands of my colleague all the attention which its great importance, as a surgical accident, demands.

But *we* also, as physicians, have much to do with hæmorrhage; with what, for distinction's sake, I may call *medical* hæmorrhage; which differs in kind, in cause, in its consequences, and in the treatment it requires, from that which surgery contemplates.

*Hæmorrhage by exhalation.*—In surgical or traumatic hæmorrhage, the blood flows from some considerable vessel, which has been cut, or torn, or somehow ruptured. You would greatly mistake, if you inferred from that circumstance (as you naturally might) that it is *usually* so—the only difference being in the situation of the vessel—in medical hæmorrhage also.

Yet that is the popular notion. When blood rushes out from internal parts, through any of the natural apertures of the body, the person is said and supposed to have broken a blood-vessel. Yet this is rarely, though it is sometimes the case. In nine instances out of ten, if there be any rupture at all, it is rupture of numerous capillaries only; but even of this there is often no evidence.

Whence, then, and how, does the blood escape from its natural channels? Why, it exudes from the unbroken surfaces of organs, without any appreciable lesion of arteries, veins, or capillaries; just in the same manner as sweat oozes from the skin, mucus from the inner surface of the bowels, and serum or synovia from the membranes that respectively furnish those fluids; and probably by the very same outlets.

This certainly is a very remarkable circumstance, if it be true; and you will naturally ask what proof we have of its truth.

The proof is simple and conclusive. We examine the surface from which the blood must have proceeded, and we find it entire; we wash and even macerate it; we employ the microscope to assist our powers of vision; yet we fail, after this careful inspection, to discover the slightest breach of substance, or any appearance of erosion.

When, for example, hæmorrhage has occurred so profusely from the stomach or bowels that the death which ensued could be sufficiently accounted for by the mere loss of blood, the whole tract of the alimentary canal has been diligently scrutinized, and has exhibited no ruptured blood-vessel, no abrasion even of its surface, nor any perceptible alteration of texture. Sometimes its mucous membrane appears, here and there, of a red colour, and, as it were, charged with blood. Sometimes it is pale and transparent, while the vascular net-work visible immediately beneath it is gorged and turgid. Sometimes the whole is colourless, the same net-work of vessels having been completely emptied by the previous hæmorrhage: and sometimes, again, (and this is very illustrative of the mode by which the blood has issued,) vast numbers of small dark-coloured masses, like grains of fine sand, can be made to start from the surface of the membrane by slight pressure. There can be no doubt that these are minute portions of blood, which had remained and coagulated in the vessels or apertures forming the ultimate channels of the hæmorrhage.

We have absolute proof, therefore, that hæmorrhage may transude through an uninjured surface: nay, in some rare cases, the process has been actually witnessed. There are well-authenticated instances on record of *cutaneous* hæmorrhage, where a dew of blood has appeared upon some portion of the skin, has been wiped away, and has re-appeared; and that again and again, without any perceptible alteration of the affected surface, beyond some occasional variation in its colour. So again the menstrual discharge has been seen to issue *guttatim* from the healthy surface of a living and inverted uterus. But I confess, that although this analogical fact helps our conception of the manner in which blood may be exhaled from an unbroken membrane, I should not lay much stress upon it for any other purpose. It is not exactly a case in point. The process of menstruation cannot be looked upon as a morbid process. During a certain portion of the life of an unpregnant female, it is not only consistent with perfect health, but even essential to it, and the fluid poured out is not strictly blood.

That the blood proceeds from the same vessels or apertures which, in health, pour out the fluids natural to the part, is rendered the more probable by this fact,—that certain hæmorrhages are ushered in and succeeded by an increased efflux of the fluids which belong to the surface concerned. In hæmorrhages from the mucous membranes, the following succession of events is, in some persons, habitual. First, there is an augmented flow of mucus alone; then of mucus tinged with blood; then of pure blood; and the hæmorrhage recedes by a similar, but inverse gradation, towards a mucous drain, which itself at length decreases or disappears.

When blood thus exudes, we say that the hæmorrhage takes place by *exhalation*. It is a convenient word, and will spare circumlocution. What the vessels or outlets to which we give the name of exhalants really are; whether they be branches from the capillaries not large enough in the natural state to admit the red particles, or whether they be mere pores in the sides of the capillaries; these are points concerning which we have no positive knowledge. We know indeed, that such channels must exist, though we cannot demonstrate or see them; and we know that while every part of the body is in a state of health and integrity, they do not allow the blood, as such, to pass through them.

Now, although internal hæmorrhage may happen in other ways, as from the bursting of an aneurism, or from an opening made in a large vessel by progressive ulceration; yet in by far the greater number of cases it takes place by exhalation. Exhalation is the rule—other modes of hæmorrhage furnish the occasional exception.

I must exclude, however, from this general statement, one very important hæmorrhage. In the brain, the former exception becomes the rule. In almost all cases, cerebral hæmorrhage results from the rupture of a blood-vessel.

There are various kinds of hæmorrhage by exhalation. I will bring them before you, in succession, as clearly and concisely as I can.

*Habitual hæmorrhages.*—In the first place, there are hæmorrhages which, although they do not belong to the state of health, if we take mankind in general, yet when they do occur, cannot properly be called diseases. There are some persons—I believe I may say there are many persons—who are subject, during the greater part of their lives, to discharges of blood; which happen again and again, commonly at regular intervals, without any perceptible detriment to the general health, independently of any obvious exciting cause, and, as it would seem, from some inherent property or necessity of the system.

Hæmorrhages thus occurring, I will call *habitual hæmorrhages.* They proceed more commonly from the rectum, or from the nares, than from any other part; although instances are recorded of their taking place from the bladder, and from the bronchi. Appertaining to the original constitution of the body, this disposition to periodic hæmorrhage has been sometimes observed to be hereditary.

You will at once be struck with the analogy which obtains between these habitual hæmorrhages occurring in either sex, and the monthly discharge which is peculiar to the female. The analogy is even closer than it may at first sight appear; but it is more distinctly marked in some individuals, liable to habitual hæmorrhage, than in others. It was one of the singular notions of the celebrated phrenologist M. Gall, founded upon this analogy, that there is such a thing as male menstruation. The points of resemblance between the two phenomena will be manifest in the following summary of the characters belonging to habitual hæmorrhage.

Like the catamenia, these hæmorrhages do not ordinarily prevail throughout the whole course of life. In most cases they do not commence before the period of adolescence; and they cease altogether, or recur at distant intervals only, in declining age. Their first eruption is sometimes preceded by a state of general indisposition, more rarely by slight febrile disturbance, and even (according to some observers) by a sort of chlorosis similar to that which affects young girls in whom the menstrual evacuation is delayed or suspended. The hæmorrhage sometimes recurs at precisely regular intervals, and by monthly periods more commonly than any other; being announced, on each occasion, by the same preludes, proceeding from the same part, continuing for the same space of time, and furnishing always about the same quantity of blood. Its accidental interruption is almost uniformly the cause or the consequence of some derangement of the health; and when it becomes excessive, it becomes, like too profuse menstruation, a disease.

*Vicarious hæmorrhages.*—It forms a very curious part of the general history of hæmorrhages, that they are not unfrequently *vicarious*, or supplemental, sometimes of each other, but more often of the monthly discharge from the uterus. Females are liable to perverted menstruation, (so to call it) through other channels than the natural one; and here again the analogy between the catamenia and habitual hæmorrhage comes into view. The hæmorrhages which belong to the constitution are apt to wander in their seat. As bleedings from the lungs, stomach, rectum, or skin, sometimes follow upon the suspension of the menses, so bleedings from the bladder, from the mouth, and from other parts, have been occasionally observed to succeed the suppression of habitual hæmorrhois.

These hæmorrhagic deviations take place commonly by the same organ on each occasion; more seldom by different organs in succession. It is almost always in this supplementary manner that the rarer forms of hæmorrhage occur, and those of the skin in particular.

This singular migration, this interchange of place between certain hæmorrhages, seems calculated to throw some light upon the obscure doctrine of *revulsion;* a doctrine to which I have already more than once referred, and which, though it is very imperfectly understood, is of frequent avail in the practice of physic.

Vicarious hæmorrhage always denotes a disordered state of the general health, and must be considered, in itself, as a malady.

*Idiopathic hæmorrhages.*—Again, there are are certain forms of hæmorrhage, not habitual, which may be denominated *idiopathic;* inasmuch as they are apt to arise without any perceptible connexion with antecedent local disease.

In other respects, however, they differ considerably, and require to be farther distinguished; and the terms *active* and *passive*, which are in common use, will sufficiently express the two forms of idiopathic hæmorrhage that I wish to bring under your notice.

*Active* hæmorrhage is preceded by active congestion, and therefore is akin to inflammation; and it often requires the treatment of inflammation.

Passive hæmorrhage often occurs without any apparent previous congestion of any kind. Hæmorrhage of this passive character has been ascribed to some change—different from that which we conceive to be produced by the distension of plethora—in the vessels or apertures through which the healthy exhalations are transmitted. The change is considered as being of the nature of morbid debility or relaxation. That such a state may sometimes exist is not impossible, nor even unlikely; but as we are altogether ignorant of the natural condition of these outlets, it is difficult to reason about the alterations to which they may be subject in disease. This hypothesis derives its chief support from the occasional efficacy of *astringent* substances (either applied locally or taken into the system) in checking the effusion of blood, when other remedies have failed.

A more probable hypothesis, perhaps, is that which supposes some alteration in the consistence or composition of the blood itself; which thus becomes attenuated, and capable of passing through channels or orifices that healthy blood, under ordinary circumstances, cannot penetrate. In support of this supposition are adduced the facts, that hæmorrhages are known to occur where the blood is more thin, pale, and serous than common; and still more remarkably where that fluid has undergone a demonstrable change in its chemical nature, or is even visibly altered in its sensible qualities—as, for example, in certain cases of purpura and sea-scurvy. And hæmorrhages of this kind are often cured by measures calculated to repair the blood; to restore it to its natural condition by improvements in diet, or by food of a peculiar kind, as the juice of lemons.

Whatever may be the true explanation of the differences in question, there can be no doubt that they exist, and are often strongly pronounced, in cases of hæmorrhage, which, inasmuch as they cannot be traced to any pre-existent local disease, we class together as *idiopathic.* And it will be worth while to run over the distinctive characters of active and passive hæmorrhage, as they are broadly and decidedly visible, in well-marked cases.

*Active hæmorrhages.* —*Active* hæmorrhage (which is preceded, I repeat, by active congestion,) occurs principally in persons who are young and robust, who live fully, and lead indolent lives, and are subject to the influence of those causes which tend to generate plethora. Occasionally the hæmorrhage can be traced to some exciting cause, such as exposure to heat, strong mental emotion, violent exercise, or bodily efforts. More frequently, perhaps, no exciting cause is apparent. It is sometimes ushered in by a set of symptoms expressive of what has been called the *molimen hæmorrhagicum.* The patient experiences a general feeling of indisposition, with wandering and obscure pains that gradually settle in the part from which the blood is about to be discharged. A series of local symptoms, such as a sensation of weight, or of tension, or of heat and tingling, sometimes a slight degree of turgescence and redness, and a visible fulness of the larger veins, indicate the afflux of blood towards the labouring organ, and the parts in its vicinity; while chilliness, paleness, and shrinking of distant parts, and especially of the feet and hands, denote an opposite state of the circulation in *them.* And to this state of things there often succeeds a general increase of heat, with a frequent, full, and bounding *pulse,*—a pulse which is so characteristic sometimes, as to have acquired a name: you may often hear, or read, of a *hæmorrhagic* pulse. The blood, when at length it breaks forth, commonly escapes with rapidity, is of a florid colour, proceeds from a single organ, and readily coagulates, though it seldom separates distinctly into serum and crassamentum. While it is flowing, the signs of local congestion diminish and disappear; warmth returns to the extremities, and the pulse regains its natural strength and frequency. The patient becomes conscious of a sensible relief, and feels stronger and more lively than before. This kind of hæmorrhage is, in some sort, its own remedy; it ceases in virtue of the discharge of a certain quantity of blood, and it is followed by morbid consequences only when that quantity has been excessive, or when it inflicts some mechanical injury upon the parts along which the blood passes.

I said that active hæmorrhage is preceded by active congestion, and is consequently akin to inflammation. Perhaps it may be more true that in some of these cases we actually have the initial stage of inflammation, of which the hæmorrhage proves the natural cure, strangling it in its birth; applying, in the very moment when it is most effective, that which, in the last lecture, I told you was the most potent of all the remedies of inflammation; namely, *loss of blood.*

*Passive hæmorrhages.*—*Passive* hæmorrhage, on the other hand, is characterized by circumstances of an exactly contrary nature. It occurs in those who are naturally feeble, or who have been debilitated by disease, fatigue, insufficient nourishment, great evacuations, or the depressing passions. It is not, in general, announced by any precursory symptoms, nor attended by any reaction. The effused blood is of a dark colour, serous, and but little disposed to coagulate; and it often is poured forth from several parts of the body at the same time. If the quantity lost be at all considerable, the natural debility of the patient is rapidly augmented; his face becomes pale, and his body loses its heat. The hæmorrhage leaves him in a worse condition than that in which it found him. The flow of a certain quantity of blood is not, as in the cases of active hæmorrhage, suspensive of its farther effusion; frequently, indeed, passive hæmorrhage resists the means opposed to it the more, in proportion as it has continued longer, or been more profuse.

*Symptomatic hæmorrhages.*—Hæmorrhages of the kind I have now been describing—that is to say, depending upon no palpable disease of any organ, and, therefore, idopathic—are of no uncommon occurrence, whether we regard the active or the passive form in which they appear: but by far the greater number of hæmorrhages by exhalation are *symptomatic;* that is, they result from some previous disease, either in the organ from which the blood proceeds, or some other organ connected therewith by some community or dependence of function.

These secondary or symptomatic hæmorrhages are preceded by congestion; but for the most part the congestion is not of the active, but of the mechanical kind, and has more to do with the veins of the part than with the arteries.

Thus we have hæmorrhage from the bronchial membrane, in consequence of crude tubercular matter in the lungs, filling up a portion of the pulmonary tissue, and obstructing the circulation of the blood through it. This is an example of symptomatic hæmorrhage by exhalation, depending upon previous disease in the organ itself from which the blood proceeds.

In some of these cases, the presence of pyrexia renders it probable that the hæmorrhage is the consequence and the relief of active congestion, provoked by the irritation of tubercles, rather than the result of a mechanical obstruction of the circulation.

Again, we have hæmorrhage into and from the lungs, as a consequence of such disease of the heart as mechanically impedes the return of the blood from the lungs to that organ,—a narrowing of the mitral orifice, for instance. Here the blood is barred up, as it were, in the lungs, till at length the capillaries, incapable of farther distension, either give way, or become so dilated as to allow of the exit of the blood through their exhalant openings, or through inorganic pores in their sides. In precisely the same way blood is poured out by the mucous membrane of the stomach and bowels, in consequence of disease in the liver, obstructing the portal circulation. These are examples of symptomatic hæmorrhage by exhalation, depending upon previous disease, not of the organ itself from which the blood proceeds, but of another organ intimately connected with the former.

When I say that hæmorrhage into and from the lungs may result from such a disease of the heart as implies an impediment to the circulation, you must not suppose that the lungs are the only channel through which the mechanical congestion can be relieved. Disease of the central moving organ of the circulation leads often, at length, to *universal* venous congestion; and the hæmorrhage, which is apt to be the consequence of such congestion, may burst forth from any part where the veins are so overloaded. Hæmorrhage from various portions of the mucous membranes, are in truth very common effects of cardiac disease.

The influence of mechanical congestion as a direct cause of hæmorrhage is sometimes very distinctly seen in the bodies of persons who have been hanged. You know that when suffocation has been produced by suddenly cutting off the access of air to the lungs; the right side of the heart, the great veins, and indeed the venous system generally, become loaded and distended with dark blood. Dr. Yelloly examined the stomachs of five men who had been executed by hanging: he found them all exceedingly vascular; and in two of the five cases, blood was actually extravasated, and adhering to the surface of the membrane: there had been, in short, unequivocal hæmorrhage.

There are several things worthy of notice, in respect to hæmorrhage by exhalation, of whatever kind.

In the first place, it occurs much more frequently and readily from some tissues of the body than from others; and most especially of all, from *mucous* surfaces. Thus we have hæmorrhage from the mucous membrane lining the nasal cavities; from the pulmonary mucous membrane; from the stomach and bowels; from the urinary organs; and from the uterus; constituting distinct forms of disease, which we are by and by to investigate more particularly. *Epistaxis*, *hæmoptysis*, *hæmatemesis*, *melæna*, *hæmorrhois*, *hæmaturia*, *menorrhagia*, are names descriptive of hæmorrhage as it is apt to occur from different parts of one or other of the three tracts of mucous membrane met with in the body; and you will find that these comprise very nearly all the complaints mentioned by nosological writers under the head of hæmorrhagy.

Now, this is a very remarkable fact, and very interesting questions arise out of it. Has it any relation to the manner in which these membranes, and the tissues subjacent to them, are supplied with a capillary circulation? Or may the fact be explained by the laxity of their attachment, which facilitates and favours the accumulation of blood in the vessels of the submucous tissue? Or has the density or consistence of their natural exhalations any thing to do with this disposition to hæmorrhage in the mucous membranes? May we suppose that the vessels or orifices appointed to exhale *mucus*, afford a more easy passage to the blood than those which give egress to thinner fluids; serum, for example, or the cutaneous perspiration? Whatever answers may be given to these questions, you will do well to recollect the fact which has suggested them.

Hæmorrhages by exhalation are not, however, exclusively confined to mucous surfaces. They are liable to occur, but much more rarely, from serous membranes. In the majority of cases, however, in which blood is found effused into any of the serous sacs, it has either been an event of inflammation, or the blood has been poured out from an accidental opening in some considerable vessel. Cutaneous hæmorrhage is also very rare, probably because the cuticle opposes a barrier to the exit of the blood; for the little red spots which characterize purpura are in fact hæmorrhages, although the blood has not penetrated the epidermis. There are cases, however, as I mentioned before, in which blood has transpired, in a sort of dew, from the external surface of the body.

Another important general fact in respect to hæmorrhages by exhalation, is, that they proceed more frequently from certain parts of the mucous membranes than others, according to differences of *age*. Thus in children they are most common from the membrane that lines the nasal cavities; in youth, from the mucous membrane of the lungs and bronchi; in the middle years of life, and towards its decline, from the rectum, uterus, and urinary organs. I should add here, from the blood-vessels of the brain, in old age; except that this, as I have already intimated, is not (speaking generally) hæmorrhage by *exhalation*.

Of course when I say that, in the instances specified, the blood is commonly poured out by exhalation, you will understand that the hæmorrhage sometimes occurs from the laying open of a single vessel of some magnitude. Thus hæmorrhage from the fauces may be the result of ulceration there, which has penetrated the coats of a vein or artery; hæmoptysis is occasionally produced by the laceration of a blood-vessel during the softening and expulsion of tubercles. Hematemesis sometimes is the consequence of the lesion of a considerable blood-vessel in the progress of cancer of the stomach, or by the extension of small corroding ulcers; hæmorrhage from the bowels is no uncommon effect of ulceration, such as happens in fever, of the mucous follicles of the small intestine; calculous matter in the kidneys will often lead to the rupture of some of the blood-vessels there, and to the discharge of blood by the urethra. Aneurisms also may burst into almost any part of the body. But events of this kind are infrequent when compared with hæmorrhage from the same parts in the way of exhalation.

In the head, however, the ratio is reversed. Blood does sometimes, I believe, exude from the *membranes* of the *brain*, but much more commonly cerebral hæmorrhage is caused by the giving way of a diseased *artery* in the brain.

How, in all these cases, to distinguish whether the blood has oozed out by many orifices from a surface, or has escaped from a hole in the sides of a vein or artery, will form matter for future inquiry. Sometimes we *can* make the distinction; and sometimes, it must be confessed, we cannot.

You will readily understand that hæmorrhage must vary greatly, in respect to its importance, and to the danger which it implies, according to the part from which it proceeds, and the circumstances under which the blood is poured out. It sometimes happens that death ensues from the mere loss of blood, either at once, by one profuse bleeding, or more slowly, by repeated bleedings which we are unable to restrain; but this is comparatively rare, and when it does happen, the blood is generally found to have proceeded from one considerable vessel, which has been ruptured or eroded. The case approximates to traumatic hæmorrhage, except that we cannot cut down upon and tie the injured vessel. Much more commonly danger arises from the presence and pressure of the extravasated blood in and upon internal parts; upon the brain, for example, in cerebral hæmorrhage,—in the lungs in pulmonary.

The symptoms also are liable to much variation in different cases. Even the diagnosis of hæmorrhage is not always equally easy, or certain. When the part into which the blood is directly poured communicates with the exterior of the body, the expulsion of some of that fluid will, generally, sooner or later, demonstrate the case to be one of hæmorrhage. I say *generally*, because cases have been known to occur, in which patients, previously in a state of great weakness, have died outright, by syncope, from the mere extravasation of the blood, and before any of it made its way out of the body. The stomach and bowels have been found full of blood, when none had passed either by vomiting or by stool. And when the blood does make its appearance outwardly, it is sometimes not easy to determine whether it has come from a certain organ, or from the parts that lie between the same organ and the natural outlet by which it ultimately escapes. For instance, it is sometimes a matter of uncertainty whether the blood, in hæmaturia, proceeds from the kidneys, or the bladder, or the urethra.

The blood itself, when it reaches the exterior, will generally be more fluid, and brighter, in proportion as it is effused in greater quantity, and nearer the surface; more in clots, and darker in colour, in proportion to the length of time that it has remained within the body after its escape from its proper vessels; and this length of time may depend upon the smallness of the quaintity of blood effused, and the consequent tolerance of the organs through which it may have passed, or upon the actual space traversed. Respecting the *colour*, however, of the effused blood, I shall have some curious explanations to offer you when I come to speak of hæmatemesis as a disease. It would be superfluous to enter upon them now.

If the site of the hæmorrhage does not communicate with the external air, we are without that certainty which results from the actual spectacle of the blood. But in such cases we are much assisted by local disturbances of function, springing from the pressure upon, or the laceration or distension of the suffering organ, or of the parts contiguous to it. And we may derive good information from observing the indirect symptoms which declare themselves through the system at large; many of which indirect symptoms are the same, whether the blood reach the exterior or not. They principally vary according to the quantity of blood poured out, and to the *rapidity* of its effusion; and some difference will occur according to the age and strength of the patient.

Some of these indirect symptoms have not always been imputed to their true cause. Paleness of the face, feebleness of the pulse, coldness of the extremities, and a tendency to syncope—symptoms which are apt to be connected with hæmorrhage—have sometimes been ascribed to the alarm and sense of danger which the sight of the blood is calculated to produce in the mind of the patient. This may, to a certain extent, be sometimes true; but the explanation cannot apply to those cases in which the hæmorrhage is strictly confined to the interior of the body, yet in which the symptoms just alluded to are often strongly marked. They *then* depend—and probably in all cases they *chiefly* depend—upon the actual abstraction of the blood from the circulation.

The management of individual cases of hæmorrhage must be mainly regulated by the particular circumstances under which they occur. The few observations that I have at present to make respecting their treatment cannot be otherwise than very general.

But a preliminary question, of some importance, presents itself. Is it in all cases of hæmorrhage proper, or safe, to attempt to stop the bleeding ?

Without going into detail, it may, I think, be laid down as a rule, that what I have called habitual hæmorrhages ought not to be interfered with, so long as they have no perceptible injurious influence upon the health, and so long as they proceed (as they mostly do) from parts, of which the structure is not likely to be spoiled, nor the function impaired, by the repeated passage of the blood. The most common seat of these habitual hæmorrhages I have stated to be the rectum ;—to which the two conditions just mentioned are, fortunately, both of them applicable. Epistaxis supplies a less frequent example of the same kind. When they deviate from their usual channel, and are transferred (as it were) to some more important organ, it will generally be right, among other remedial measures, to endeavour to recall the original hæmorrhage. It is very seldom that the metastasis takes place *for the better*,—i. e. from a part where the bleeding is attended with danger, to one where it is comparatively harmless.

However, when these habitual hæmorrhages happen, as they often do, in plethoric persons; and when they are urged and kept up, as they frequently are, by intemperate and luxurious habits; we ought not to content ourselves with merely looking on. Hæmorrhoïs often performs the office of a safety valve in such persons; and there are many who have what are called bleeding piles, and who would rather continue to have them, than submit to any change in their mode of life, or to the employment of other means of evacuation. Certainly these are cases in which nothing should be done to stop the bleeding; yet such patients ought to be told that the hæmorrhoidal discharge is but a precarious, and often an inadequate relief of the plethora: that while the plethora is suffered to exist there is danger of a cessation of the piles, and of the supervention of serious or fatal affections of other parts, and especially of the head. Apoplexy, or cerebral hæmorrhage, has frequently been known to follow hard upon the suspension of constitutional hemorrhoïs. The patients should be admonished also that the discharge of blood from the vessels of the rectum may become excessive; that if it be aggravated by exercise, or in any other way, it may lead to inflammation about the anus, and to great inconvenience; and that there are safe and tolerably sure methods of getting rid of the plethora, (which is what chiefly constitutes the danger of such cases,) if they will submit to the observance of them. It is in the intervals between the hæmorrhages that the danger of which they are in some sort the token, may best be met.

Again, it will seldom be proper to employ *direct* expedients for stanching the flow of blood, in the small class of active idiopathic hæmorrhages; unless the quantity lost is so great as to endanger the safety of the patient. Such hæmorrhages have commonly a tendency to cure themselves, by relieving the general plethora, or the local congestion, on which they depend. For these hæmorrhages, which bear so strong an analogy to inflammation, the *treatment* of inflammation may often be requisite, as an indirect mode in which their amount may be moderated, and their recurrence obviated.

With these exceptions, both direct and indirect measures are to be used, for arresting the effusion of blood as speedily as may be.

To this end the patient is to be surrounded as much as possible with cool fresh air, and kept in a state of absolute quiet. All motion of the body, and emotion of the mind, all kinds of stimulating food and drink, every thing, in short, which has a tendency to hurry the circulation, should be diligently avoided; and that position of the body should be chosen which is the least favourable to the afflux of blood towards the part affected. The horizontal posture will be proper in hæmorrhage from the bowels, the uterus, or the urinary organs. In epistaxis, and in cerebral hæmorrhage, the head should be raised.

In two words, the *antiphlogistic regimen* should be strictly enjoined in all cases of hæmorrhage sufficiently severe to require medical assistance.

Of the actual remedies used for checking the farther escape of the blood, one of the most important has already been alluded to—I mean venesection. We are guilty of homœopathy in this matter: to prevent bleeding, we draw blood. After what was stated respecting the use of blood-letting in inflammation, I need not dwell upon the objects aimed at by this measure: they are, briefly, to abate the vigour and force of the heart's contraction, to lessen general plethora when it exists, to remove local congestion, and to divert the current of the blood from the suffering organ. The method, and the amount, and the repetition of the blood-letting, must of course be regulated by the circumstances of each particular case. And the same objects may sometimes be effected by other modes of general depletion, especially by the use of purgative medicines.

Next to blood-letting, *astringents* constitute the great resource against actually existing hæmorrhage: and among these, *cold* is one of the chief. It may be placed in direct contact with the bleeding surface:—as when ice is *swallowed* to restrain hæmatemesis: or cold water injected into the rectum in excessive and exhausting hæmorrhoïs; or into the vagina, in flooding from the uterus. Or it may be applied to the surface of the body, as near as possible to the seat of the hæmorrhage; as to the nose and forhead in epistaxis; to the chest in hæmoptysis; to the epigastrium in hæmorrhage from the stomach; to the lower part of the abdomen or to the perinæum in hæmorrhage from the intestines, uterus, or urinary organs. But the influence of cold in constringing the smaller vessels is not confined to the part with which it is in contact; it will stop hæmorrhage by the sympathetic shrinking which it produces in distant parts. Epistaxis, for example, has often been arrested by the sudden apposition of cold water to the neck, back, or genital organs. The nursery remedy consists in slipping a cold key down the back betweeen the clothes and the skin.

Of even the mischievous power of cold in this way we have continual illustration of the suppression of the catamenia by cold and wet accidentally applied to to the feet.

There is a long catalogue of medicinal substances which are esteemed to possess more or less of a specific virtue, when taken internally, in checking the flow of blood. Most of these are of an astringent nature, and some of them are eminently useful. The acetate of lead enjoys, in this country, a higher character, perhaps, than any other of these substances.

Many vegetable matters, and some artificial compounds, frequently employed in internal hæmorrhages, seem to owe their astringent and styptic properties to the gallic acid which enters into their composition. Such are the rhatany root, uva ursa, bistort, tormentil, the pomegranate, kino catechu, the several preparations of gall-nuts, and the nostrum called *Ruspini's styptic.*

The power of arresting internal hæmorrhage has also been confidently ascribed, by different persons, to nitre given in large doses, to the mineral acids, to the muriated tincture of iron, to alum, to the oil of turpentine, to the secale cornutum or spurred rye, and to various other substances; a more particular account of the rules and indications for administering which, I may return to, when I have to speak of individual hæmorrhages.—*London Medical Gazette.*

---

Sir Benjamin C. Brodie *on Mortification from Strangulation or Ligature.*

A ligature drawn round any part of the body, so as to intercept the communication with the great vessels and the heart, may cause that part to perish. But the effect of the ligature is not the same in all cases; and it does not always produce mortification in the same way. You apply a bandage round the arm before you bleed a patient, to make the veins of the forearm become distended, the object being merely to stop the circulation in the superficial veins. If you take it off at the end of a few minutes, the hand is at once just as it was before the ligature was applied. If you were to leave it on for twelve hours, the whole hand and forearm would become swollen, and would remain swollen for some time after the bandage was removed. The swelling in such a case arises from the congested state of the veins, and from the consequent effusion of some of the serum of the blood into the cellular membrane. If the ligature round the arm be still tighter, so as to obstruct the circulation to a greater extent, but without arresting it altogether, the same effect is produced, namely, serous effusion, which may continue for some time after the cause which produced it is taken away. The first effect, then, of a ligature which obstructs the circulation without arresting it completely, is to produce serous infiltration of the cellular membrane, and an œdematous swelling. The different kinds of dropsy depend on the same principle. Disease in the heart, impeding the circulation through it, gives rise to anasarca of the legs, and dropsy of the pericardium and pleura. Disease of the liver produces dropsy of the peritonæum.

But let us suppose that a ligature is applied in this manner round the arm, and allowed to remain, so that the impediment to the circulation continues. A low sort of inflammation is set up, the œdematous swelling and the tension are aggravated, and this may terminate in mortification.

This is one kind of mortification from ligature. But let us suppose that the ligature is drawn tighter still: that it completely intercepts not only the venous, but the arterial circulation. It is evident that the part below the ligature, being altogether deprived of that supply of scarlet blood which is necessary to the maintenance of vitality, must lose its vitality; and this, then, is another way in which a ligature produces mortification.

In the course of your practice you will meet with numerous cases illustrative of the different effects of ligatures according to the degree of constriction which they occasion. Thus, a woman has a femoral hernia. A large portion of intestine is protruded through the narrow crural ring in the act of coughing. The ligature is as tight as possible. The strangulation complete. The arterial circulation as well as the venous is completely obstructed. If you perform the operation for strangulated hernia on such a patient, even in half an hour, you may find the intestine dead. But if (as generally happens) the degree of constriction is less, in consequence of the opening being larger, or the protruded intestine being smaller in quantity, then the venous circulation is obstructed more than the arterial; there is no mortification immediately; there is venous congestion, followed by inflammation, which may end in mortification in the course of two or three days, or, perhaps, not until after the lapse of a longer period. A man has a phimosis. He pulls back the præpuce, and the orifice becomes a stricture behind the *corona glandis.* There is venous congestion. The glans is swollen, assumes a purple colour, then becomes exceedingly inflamed, and that inflammation is followed by mortification. Again, a patient has internal piles. They protrude at the anus; the sphincter muscle acts spasmodically upon them. They cannot be pushed back through the sphincter: the return of venous blood is prevented; they swell, inflame, and, in the course of a few days, they mortify. By and by the slough drops off, and the disease is cured.

You will now understand the principle which ought to be kept in view when we use ligatures in surgical operations. You cure internal piles by a ligature. If you draw the ligature only moderately tight, you do not kill them at once: they swell: they inflame: they may die at last, but not till after a painful and tedious process. But if the ligature be drawn as tight as possible, it stops the flow of the arterial as well as of the venous blood, and the piles die directly. This is the way in which a ligature should be applied in almost all cases of surgical operation: it should be drawn as tight as possible. In dealing with piles, or nævi, or tumors of the tongue, the tighter you draw the ligature the sooner the sufferings of the patient are over. If you do not draw it tight, he suffers for a very long time, and very greatly; nay, perhaps severe constitutional symptoms may ensue.

I have said that when you apply a ligature in a surgical operation, your object should be to stop the flow of arterial blood at once; and you might suppose that if the ligature were kept on for half an hour, or an hour, that that would be sufficient; that the part being deprived of the flow of arterial blood for such a time it would certainly lose its vitality. But this is not exactly the case. You apply a ligature round an artery, draw it as tight as you can; it divides the middle and inner coats, but only compresses the outer coat. It makes a slough of a little piece of the latter; and when the ligature comes away at the end of ten days or a fortnight, you find the slough in it. But if you cut off the ligature in half an hour, or an hour—an experiment which has frequently been made—there is no slough. The artery may be obliterated, or it may not, by the effusion of lymph; but the piece of the outer coat that was included in the ligature recovers itself; at least it does not come away as a slough. I once had occasion to observe the same thing illustrated on a larger scale. I had a patient with a malignant tumor of the tongue, which, according to the method suggested by Sir Everard Home, I determined to remove by ligature. I drew the double ligature as tight as I could; and when I saw the patient half an hour afterwards, the piece of the tongue included in the ligatures was quite livid, and apparently dead. I saw him again in three or four hours, and found him suffering a great deal of pain and inconvenience. It occurred to me that the piece of the tongue had been dead for some time, and that I should, perhaps, give relief by cutting off the ligature. With some little trouble I succeeded, but, to my great annoyance, the next day I found the whole piece, which appeared to be dead, alive again. The ligature, therefore, in surgical operations, must be drawn as tight as possible, and then left on until it is separated by a natural process.

*Mortification from pressure.*—Parts may be killed by pressure. The mode of death here is nearly the same as when parts are killed by ligature. The difference being simply this: the pressure is like a ligature applied to a broad surface, operating not on the arterial and venous trunks, but on all the small vessels and capillaries. Mortification from pressure is chiefly observable when the pressure is made on parts which lie over a bone; where there is no cushion of flesh between the skin and the bone. If the pressure be very tight, it may produce mortification immediately. I remember that when I was a student, a man came into the hospital with a fracture of the leg. The surgeon applied splints, and drew a bandage over them round the foot as tight as possible. The next day the man was in a great deal of pain and suffering. The bandage was removed, but it had already occasioned a broad slough of the skin over the instep. I have in other instances seen sloughs produced in the same manner, as it were instantaneously, in consequence of bandages being applied too tight.

But in the great number of cases where mortification is the result of pressure, it does not occur immediately, but after the lapse of some time; and it is not a direct, but a secondary consequence of the pressure. A man, for instance, is bed-ridden; he lies on a hard mattrass; he becomes very thin; the skin over the os sacrum becomes tender to the touch, it inflames, assuming a dark red colour; vesications form upon it; the inflammation goes on, and ends in mortification. Hence, though pressure may produce immediate mortification in some instances, yet in ordinary cases it does so by causing inflammation first, which inflammation, the pressure being continued, ends in the same manner.

This kind of mortification from pressure takes place unden certain circumstances more readily than under others. A man is weakened by continued fever, and, from the state of debility in which he then is, pressure on the skin over the os sacrum and other projecting parts of bone will produce mortification, while it would not produce it if he were in vigour and in health. After injuries of the spinal cord, mortification from pressure is very readily induced. A man has the spinal cord torn through in the middle of the back; and you find, almost before you suspect that there is any thing wrong, a great slough over the sacrum. Nay, the pressure of the mattrass against the ankles will, in such cases, produce mortification. I have known mortification begin in the ankle within twenty-four hours after an injury of the spine; and a remarkable circumstance it seems to be, that injuries of the spinal cord should thus lessen the vital powers, so as to make the patient liable to mortification, when we consider how many circumstances there are that would lead us to doubt whether the nerves have any influence over the capillary circulation. The circulation, viewed by a microscope, in a frog's foot, goes on just the same whether the nerves are divided or not. In an experiment which I was making on poisons, I divided all the nerves in a dog's axilla; I then divided all the skin which was attached to the anterior extremity, and then the muscles and cellular membrane, so that there was an absolute want of union between the extremity and the trunk, except by means of the axillary artery and vein, which I left untouched. The animal, at the expiration of twenty-four hours, was killed; but the limb maintained its vitality perfectly all the time. In spite, however, of this and of other circumstances which I might mention of the same kind, a concussion of the spinal marrow makes the patient liable—and sometimes almost immediately—to mortification of the parts below.

Patients are more or less liable to mortification from pressure, accordingly as they are more or less emaciated. A man with a good cushion of fat between the skin and the os sacrum, or the skin and the great trochanter, is not so liable to the formation of sloughs in those parts as a thin one; and that for obvious reasons.

When you suspect that pressure on any part is so great as to be likely to occasion mortification, you can do nothing but remove the pressure. When a bandage is placed in a case of fracture, you must remove it as soon as you suspect that the swelling of the parts has made it very tight, lest mortification should follow. When a patient has so long been confined to his bed, that you expect mortification will take place, you must endeavour to guard against it. It is more easy to prevent it than to stop it when it has once begun. How, then, is this to be accomplished? If a patient lies on his back, the skin sloughs over the os sacrum; if on one side, then it sloughs over the great trochanter. Endeavour, when he can manage it, to make a patient vary his posture. If he can be shifted, let him lie at one time on his back; at another, on his side: nay, let him turn round, and lie occasionally on his face. If you have what they call a prone couch, properly constructed for the purpose, he may, in many instances, use it to great advantage. In one of the worst cases of this kind, when mortification had begun, I used to turn the patient on his face many hours in the day, and with perfect success. But sometimes the patient cannot be shifted. There may be fracture of the thigh, and he must lie always on his back. You must then endeavour to take off the pressure by other means—by an air cushion with a hole in the centre, the tender part over the os sacrum being in the hole of the cushion. But in all cases where you use an expedient of this kind, you should first apply a piece of common soap plaister, spread on calico, over the part, to support it. If you merely place the hole of the cushion under the os sacrum, the skin will bulge into the hole, and the patient will lie as bad as if there were no hole at all, or even worse. The same rule applies to all cases where you use contrivances to take off pressure, as in those of corns and bunnions. In cases where you can have recourse to it, the water-bed is very useful in preventing mortification from pressure. Dr. Arnott's hydrostatic or water-bed diffuses the pressure evere where. When you lie on a mattress, the pressure is thrown on all the prominent parts of the body, and little elsewhere; but in using the water-bed the water rises to fill up the hollow places, and the pressure is not greater on the sacrum than on other parts. No doubt this bed is the best method which has yet been contrived for preventing mortification from pressure—the only objection to it is, that it is not applicable to all cases. In cases of compound fracture of the thigh or leg, for example, it would not give sufficient steadiness to the injured limb.

But another plan may be adopted to prevent mortification from pressure—that is, to prevent the inflammation which precedes it. The thicker the cuticle the more it will protect the parts beneath it. You may, if you attend to it in time, add to the thickness of that cuticle by stimulating the surface of the skin. Nurses know this very well, for when patients are bed-ridden they wash the parts subjected to pressure with brandy. What is better, is a lotion composed of two grains of the oxymuriate of mercury to an ounce of proof spirits. When you think that a patient is likely to be confined so long in bed that there may be mortification from pressure, wash the parts two or three times a day with this lotion. I have found it useful in other cases where a patient suffers from pressure. A man has a rupture which requires to be supported by a very powerful truss. It galls and frets the skin, and may at last cause inflammation and sloughing; but under the use of the lotion, a thicker cuticle is generated, and this mischief is avoided.

The sores which remain after the separation of a slough produced by pressure, are to be treated like common sores; this being kept in view, that the skin will slough again if the pressure be continued. You must, if possible, contrive to take the pressure off these sores; but, unfortunately, it is not always possible for you to do so, and in spite of all your care and trouble slough will form after slough, exposing the sacrum or trochanter, or other bony structures, whatever they may be.

*Mortifications from contusions and traumatic gangrene.*—I now come to speak of mortification from a blow or other mechanical severe injury. It may be said that pressure is mechanical injury, but I now speak of a sudden injury operating for a short space of time, such as a contusion or a wound. The effect of mechanical injury may be to produce mortification, which is confined to the parts actually injured. For instance, a man gets a kick on the shin, and the next day there is a slough, and the skin is dead, just where he was kicked. Why? Because the kick bruised the skin against the bone, ruptured the capillary vessels, and destroyed the organization in the part, so that life could not go on. But here the mortification is confined to the part actually injured. A remarksble circumstance happens in some of these cases. The cellular membrane has not so much vitality as the skin, and therefore perishes more easily. A blow will disorganize the cellular membrane which will not disorganize the skin. A man came into the hospital who had had a severe blow on the instep; there was a purple appearance, but not very extensive ecchymosis, and I thought nothing of it. The next day I found the part inflamed, the following day there was a good deal of swelling, and on the third day the skin was beginning to slough. I divided the skin with a lancet, and found a large slough of the cellular membrane. The blow had pressed the skin and cellular membrane against the bones of the instep, and had killed the latter but not the former. The slough of the cellular membrane would have been followed by an extensive sloughing of the skin, if acting on the principle explained in my last lecture, I had not divided the latter freely. In cases in which you suspect that the cellular membrane may be destroyed while the skin is not, you must watch the patient, and if there be swelling and inflammation you should divide the skin, and save it from perishing as far as you can, though you cannot save it entirely.

But in other cases the mortification is not confined to the part actually injured, but may extend to the greater part of the limb. These are the cases to which the name of traumatic gangrene is applied. A man sustains a severe injury in the leg, and a great part of it mortifies. It would appear that the mode in which traumatic gangrene is produced varies in different cases. Mr. Guthrie, for example, describes a case in which mortification of the leg took place as high up as the knee, in consequence of a blow on the back of the leg. The limb was amputated, and when he came to dissect the parts it was found that the blow had lacerated the lining membrane of the popliteal artery, in consequence of which there had been effusion of lymph into the cavity of that vessel, stopping it up. That alone might not have produced mortification, but the anterior and posterior tibial arteries were torn through also, and the result of this double injury was that the limb, not getting a proper supply of blood, perished. In this case the pressure of extravasated blood might have contributed, in some degree, to produce the mortification. But local extravasation of blood, if it exist to a great extent, is, of itself, sufficient to produce this effect. When I was house-surgeon a man was brought into this hospital with some kind of tumour about the groin, but no pulsation was felt in it, and no one suspected that it was an aneurism. There was severe pain felt in the thigh, evidently arising from pressure on the anterior crural nerve, and the event proved that there was an aneurism, though it had not been indicated by the usual signs. It burst one day into the cellular membrane; the man screamed out as if he was being murdered, so horrible was the pain. The next day there was gangrene as high up as the groin, and the man died in about a fortnight. On dissection we discovered an aneurism of the internal iliac artery, which had burst under Poupart's ligament. The extravasation of blood had prevented the circulation from being carried on in the limb, and hence it mortified. There was a man in the hospital, long ago, who had popliteal aneurism. I had fixed the day for tying the femoral artery; but on the day previous to this, the aneurism burst into the calf of the leg, and the next day the limb was in a state of mortification; so that instead of tying the artery I amputated the leg. The vessels below were all quite pervious, and the circulation would have gone on very well but for the pressure produced upon them by the immense extravasation of blood. No doubt, in many cases of traumatic gangrene, this is one principal cause of the mortification.

But traumatic gangrene takes place in another way, and, to illustrate what I mean, I will mention the circumstances of a case which occurred in the hospital some few years since. A poor boy was jumping over a ditch, and came with considerable force upon his feet. There was a compound fracture of the leg above the ankle. The external wound was trifling, but it was evident that a great shock had been given to the foot and the leg. Four days afterwards the limb was in a state of mortification as high as the knee, and the mortification seemed to be extending to the thigh. I amputed the thigh as high up as I could, near to the great trochanter. We dissected them very carefully. The large arteries, and all the large veins, were quite pervious. There was, in fact, no injury whatever to the arterial trunks: but the cellular membrane, the muscles, and, in short, all the structures, seemed to be more or less disorganised. There were spots of ecchymosis in the large nerves; the periosteum was universally detached from the fibula, and very nearly so from the tibia. How does the periosteum adhere to the bone? By the small vessels. It is evident, then, that the shock of the accident must have occasioned a great injury to the small vessels connecting the periosteum to the tibia and fibula, and the probability is, that the same kind of injury inflicted on all the capillary vessels of the limb laid the foundation for the mortification. I do not see how the occurrence of mortification in cases like this can otherwise be explained.

It has been a sort of *dictum* of the schools of surgery, that you should not amputate while mortification is going on; and certainly, when there is mortification from ossified arteries (as I shall hereafter explain,) or where there is mortification from inflammation, you ought to wait for the mortification being stopped, and for the formation of a distinct line of separation, before you proceed to an operation. But it must have been palpable to every body who took the pains to consider the subject, that this rule would not apply to all cases of mortification.

For example, a man has a strangulated hernia; when you open the sac you find the omentum strangulated, a part of it dead, and the mortification still extending. You would not hesitate in a case like this to cut off the dead and dying omentum. If piles were undergoing the process of mortification from being strangulated by the sphincter muscle, you would not hesitate to cut them off. You may conceive many other cases, in which the cause of mortification is local, and to which the general rule which I have just mentioned does not apply. Baron Larrey has the credit of having pointed out more distinctly than had been done before, that where there is mortification from local injury you may venture to amputate, though the mortification is still spreading. But I apprehend that the operation must be had recourse to at once, and that the case admits of no delay. If in g nsequence of local injury to a limb, mortification has begun, but has not yet produced any severe shock on the system, there you may amputate. But where the mortification has been going on for some days, so that the system has begun to be influenced by it, the pulse getting weak, perhaps intermitting, and with great prostration of strength, in such a case you must not venture to amputate. Under such circumstances it is probable that the system is not in a state to bear the additional shock of the operation. However, I believe that cases enough may be adduced to prove that Baron Larrey's rule of not waiting to amputate till the mortification has stopped, is applicable in a great nnmber of instances where the disease arises from local injury. It is good in theory, and there is now sufficient experience to enable us to say that it is good in practice also.—*Ibid.*

---

*Tetanus cured by Tobacco.* By H. BULLOCK, M. D.—George Clarke, aged 33, a healthy labourer, of regular habits, was admitted as a patient of the Uxbridge Dispensary, labouring under symptoms of tetanus, on Thursday, the 13th of May. Upon entering his apartment, I found him lying in a state of opisthotonos, with his countenance peculiarly characteristic of the disease, the paroxyms occurring about once in three minutes, when the pain was most agonizing, whilst during the interval even the muscular rigidity was intense, the perspirations profuse; the function of deglutition was performed with the utmost difficulty, and never without producing a spasm.

The history of the case, as obtained from the patient and his wife, is, that six weeks ago he received a blow on the left side, between the lower rib and the crista of the ilium, and that he has had pain in that situation ever since, though not sufficient to incapacitate him for his ordinary occupation of a labourer until Tuesday, (two days before I saw him,) when he complained of stiff neck and sore throat, with an almost entire inability to swallow, which symptoms have increased up to the present time. His pulse is 80, and regular. Firm pressure over the seat of the accident creates no further pain than when applied to any other portion of the abdominal muscles, which always has the effect of exciting a paroxysm; nor is there any perceptible fulness there. The trismus admits of the separation of the jaws to an extent of about the third of an inch. His respiration is hurried, and he describes a constant and severe pain in the site of the diaphragm, extending from the ensiform cartilage to the spine. I now prescribed—

℞ Hyd. Chlor., Ext. Coloc. Co. aa. gr. v.; Ol. Croton. ♏j. ft, pil. ij. st. sumend.

℞ Tabaci Foliorum, ℥ss.; Aquæ ferventis, ℥ix. Macera per quartam horæ partem, et cola ft. enema statim injiciend.

*Evening.* The remedies have had the desired effect of removing from his bowels large, black, and scybalous evacuations. There had been no collapse peculiar to tobacco, and the man's sufferings appeared rather enhanced than alleviated since my visit in the morning.

Enema repetatur sextâ quâque horâ.

℞ Quiniæ Disulph., Ferri Sulph., Zinci. Sulph. aa. gr. ij.; Acid. Sulph. Dil. ♏v. ex Mist. Camph. 3tiâ quâque horâ. Brandy in arrow-root, and strong beef-tea.

I will not render the perusal of the case wearisome, by a narration of the daily reports and slighter signs of amendment and alterations, but will merely state the general outline. The above plan having been rigidly adhered to for three days, the patient had very materially improved, the paroxysms occurring about every twenty minutes, when they were shorter in duration and less in severity. There is great permanent rigidity of the whole muscular system. The trismus has so much diminished as that the first joint of the index finger can be now easily admitted into the mouth; there is yet extensive opisthotonos; his countenance has very much improved, his perspirations lessened, his breathing is not so much oppressed, and he is now able to sleep for half an hour at at a time: his pulse remains the same, except at the period of a paroxysm, when, of course, it becomes disturbed; however, this shortly subsides. The tobacco produced the usual symptoms of excessive prostration every time it was administered, which generally continued for two hours, with the exception of the first three, when there was no sensible effect. This agent was now omitted, and he was to continue the tonic with half a pint of brandy per diem, and

Hydr. Chlor., Extr. Coloc. Co. aa. gr. v. ft. pil. ij. alt. nocte.

5th day.—Since the last report[illegible]at of the third day, all the symptoms have returned, and in a more aggravated form than previously, the jaws being perfectly clenched, and his pulse quick and feeble. Notwithstanding this depressed state of the circulation, the enemeta were again ordered, and to be injected every eight hours; and after the two first he began to improve; if, however, from any accident, a longer interval were allowed to elapse, the spasm and profuse perspiration immediately increased. This treatment, omitting the pills, was diligently persevered in for four days, without intermission, and with the best effect; then the tobacco was discontinued gradually.

12th day.—He is now very much better; indeed, there is scarcely any spasm, and he is able to open his mouth without restraint. He was desired to continue his medicine three times a day, with calomel and colocynth every alternate day. Port wine, a pint daily, ale and mutton chops.

21st day.—The man is quite well, and able to walk out, merely suffering from debility. Mr. James, who witnessed the case throughout, also observed the very remarkable relapse on the omission of the tobacco, and the immediate amendment on its renewal.

I have termed this case one of idiopathic tetanus, merely because it is not one of visible traumatic. From the fact of there having been pain in the situation of the blow ever since its infliction, I am disposed to think some injury was sustained, whether by nervous filament, muscular fibre, or fascia, is uncertain; but probably there was partial rupture of one or more tissues, which I apprehend in a peculiar state of constitution would be inadequate to excite the violent form of reflected constitutional irritation which we designate tetanus. But the treatment is what I am anxious should attract particular attention. I am aware I have no new remedy to suggest; indeed, I should search the Pharmacopœia in vain; were I to attempt to find a remedy that had not been employed in the treatment of tetanus. Tobacco, then, I consider our sheet anchor in arresting this formidable disease; but it must be employed with a decision and boldness commensurate only with the fatality of the malady, and the extreme urgency of the case. The disease being so rapidly destructive, our time and field of action are very limited; hence I have been induced to use this desperate remedy with a fearless perseverance, as possessing the peculiar property of most powerfully affecting the *entire* system, desiring at the same time to fortify the nervous system by tonics and stimulants; also to introduce as much support as possible by means of the strongest animal broths and jellies, for the effect of the remedy, I conceive, as well as that of the disease, upon the vital powers, would tend to render the support imperative; nor is there any thing incompatible in this mode of treatment.

It is worthy of remark that the patient has never experienced any of the original pain in the side since the appearance of the tetanic symptoms.—*Ibid.*

---

*Compound Nature of some of the supposed elementary Substances. Conversion of Carbon into Silicon.*—The Edinburgh savans have, during the last few weeks, been labouring under considerable excitement in consequence of the alleged discoveries of a young physician of that city; which discoveries, if true, will create a complete revolution in chemistry, as well as in all the allied science. It was reported that a Dr. Brown had positively succeeded in converting carbon into silicon, and that Professor Christison had repeated his experiments, and found them to be correct!! Furthermore it was stated that Dr. Brown was occupied with the transmutation of other supposed elementary substances, and that he had succeeded in converting iron into rhodium; and we understand that specimens of the metals have been publicly exhibited, though the process for effecting the transmutation has not yet been published. Rumor also reports the conversion of oxygen into sulphur, magnesium into calcium, and the decomposition of arsenicum.*

For several years past chemists have been acquainted with the fact that two or even three substances, possessing very dissimilar physical and chemical properties, may be composed of the same elements, and in the same relative quantity. Thus cyanogen gas is a compound of—

| | | |
|---|---|---|
| 2 atoms Carbon | . = | 12 |
| 1 atom Nitrogen | . = | 14 |
| 1 atom Cyanogen | . = | 26 |

And paracyanogen, a brown solid, is composed (according to Professor Johnstone,) of—

| | | |
|---|---|---|
| 8 atoms Carbon | . = | 48 |
| 4 atoms Nitrogen | . = | 56 |
| 1 atom Paracyanogen | = | 104 |

Now 12 : 14 : : 48 : 56. So that the relative proportions of carbon and nitrogen in these two bodies is the same. Such substances are called *isomeric.*

On the 15th of February, 1840, a paper

* By the way, Dr. Lambe (Medical and Experimental Inquiry into the Origin, Symptoms, and Cure of Constitutional Diseases, London, 1805,) long since asserted that arsenious acid was generated by the putrefaction of organic matter; and in 1828 he announced (An Investigation of the Properties of Thames Water, p. 14,) that arsenicum (or a substance simulating it) had been procured from Thames water. The recent assertion of Orfila, that arsenicum exists in the human bones, (a statement the accuracy of which Dr. O. Rees has lately denied,) and the alleged discovery of Dr. Brown, gives additional interest to the statement of Dr. Lambe.

was read to the Royal Society of Edinburgh—

*On the Preparation of Paracyanogen; and on the isomerism of Cyanogen and Paracyanogen.* By Samuel M. Brown, M. D.*—In this memoir, the author, after making some critical observations on the history of the discovery of the remarkable body, paracyanogen, proved by a selection of experiments, that, once formed, it can never be resolved into cyanogen again, and made it evident that the cyanogen obtained from it by Professor Johnstone (Phil. Trans. 1838,) had only been retained by the absorptive power of paracyanogen. The main object of the paper, however, was to make out, by a series of experiments, that *the bicyanuret of mercury, decomposed under high pressures, is resolved into mercury and paracyanogen;* and in the course of the experimental details, Dr. Brown described a "different barometer," which he used to estimate the degree of pressure (1.78) under which cyanogen is separated from mercury as paracyanogen.

Perhaps the most interesting part of this novel contribution to the science of chemistry, was that which contained the author's view of the constitution of paracyanogen, and of isomeric groups of bodies in general. He represents paracyanogen as a cyanide of cyanogen, *Cy* and *Cy*, a compound of two " equal and similar atoms." Our space does not admit of a statement of his argument; suffice it, that once established, it deeply affects the doctrines of affinity and constitution which have hitherto been laid down, and affords a practical foundation for an hypothesis of the composition of the elements, susceptible either of establishment or refutation by experiment.

Professor Forbes having stated the mathematical difficulty which stands in the way of the conception of the chemical union of two "equal and similar atoms," Dr. Brown explained that the perception of that very difficulty on his part, had been the mental initiative of his experiments.

We understand that this memoir is to be followed by another, on the transmutation of carbon into silicon, or, at all events, of some one element into some other.

The following is a notice of Dr. Brown's paper, read at the Royal Society of Edinburgh, on the 3d of May, 1841, on the conversion of carbon into silicon, given in Sir W. Jardine's Annals and Magazine of Natural History for June.

*Experimental Researches on the Production of Silicon from Paracyanogen.* By J. Brown, M. D.—The author had intimated in a former paper, that he had been led to infer from experiment, that two familiar substances, long and universally regarded as distinct elements, are only modifications of one and the same material form; and having extended his inquiries, he now ventures to maintain that carbon and silicon are isomeric bodies. The method in which he establishes this proposition is very simple, and consists in the description of a number of processes by which carbon may be transferred into silicon; and crucial experiments, intend to prove that there is no intelligible source of fallacy in the processes which are given. Accordingly, the present communication is of a freely practical character. It is composed of five sections: the first treats of the production of silicon from free paracyanogen; the second of the formation of amorphous, mixed siliciurets of copper, iron, and platinum, by the reaction of paracyanogen on these metals; the third, of the quantity of nitrogen separated from paracyanogen when it is changed into nitrogen and silicon; the fourth describes processes for the preparation of definite and crystalline siliciurets of iron from the paracyanide of iron, and from the ferrocyanide of potassium; and the fifth gives easy formulæ for the extraction of silicic acid from the ferrocyanide of potassium by the action of carbonate of potassa.

Our reason for noticing this investigation in a periodical devoted to the objects of natural history, is this: if Dr. Brown's observations be corroborated by those who repeat his singular experiments, there will be opened up a new sphere of geological inquiry of the highest order. With the aid of a transelemental chemistry (for we understand Dr. Brown has transformed several other elementary forms besides carbon,) we may approach the subject of the molecular genesis of the earth; and the geological relations of carbon and silicon are certainly sufficiently striking to warrant the entertainment of this hope. As it is, there are several points in natural history which seem to be illustrated by the particular case of transformation now in hand. As one instance, we could specify the siliceous character of many organic remains, found in circumstances in which the source of silicon is perplexing and unintelligible.

In the discussion which followed, Professor Traill remarked, that though he had not had an opportunity of repeating Dr. Brown's experiments, yet, from his acquaintance with that gentleman, he had a strong conviction of their value; and this notwithstanding the very startling principles and extraordinary conclusions to which they necessarily led. He had no hesitation in saying, that since the early days of Davy, when that great chemist brought the metalloids to light, no investigation had been made approximating in importance to the present, whose publication would do honour to the Society, and whose interest, as it regarded the subjects of Botany, Palæontology, and Geology, in its widest range, was altogether unbounded.

* Edinburgh Monthly Journal of Medical Science, March, 1841.

Professor Christison begged to meet a statement which he had understood had gone abroad, that he had given a guarantee to the accuracy of these investigations. This was by no means the case. The fact was, that, now for some time otherwise employed, he was not capable of testing these admirable experiments: no one, in fact, could do so but a first-rate analytic chemist, perfectly master of the most recent manipulations of the laboratory; and he would warn every one against coming to a decision regarding these conclusions, well styled *startling*, either for or against such preliminary investigation. At the same time, it was true that he had been familiar with the details of the inquiry; he had searched, along with the author, but in vain, for grounds of fallacy, and he formed the very highest estimate of their value and importance.

As some of our chemical readers may, perhaps, be desirous of submitting Dr. Brown's statements to the test of experiments, we subjoin the following directions for the preparation of silicic acid from ferrocyanide of potassium.

"Mix equal weights of anhydrous ferrocyanide of potassium and carbonate of potassa, and keep the mixture five hours (for 2000 grains of the ferrocyanide) at a good white heat, in a well closed crucible of hammered iron. The product yields to water a mixed solution of cyanide of potassium, undecomposed carbonate of potassa, and silicate of potassa. The silicic acid may be separated by adding an excess of hydrochloric acid, ignition, and elutriation."

It would be premature at present to criticise Dr. Brown's paper, inasmuch as its accuracy must be established or disproved by experiment, and not by mere reasoning. We regret much that in the reports of it hitherto published no mention is made of the quantity of silicic acid obtained from a given weight of ingredients used; because if the quantity be relatively small, the probability is that the conclusions of the author are erroneous; for the obvious sources of small quantities of silicic acid in the above experiments would be the glass vessel (presumed to be) used in some of the processes, the iron crucible, the carbonate of potassa of commerce, and the ferrocyanide of potassium.—*Ibid.*

---

*Dislocation of the Thumb.*—At the last meeting of the Hunterian Society on the 9th of June, Mr. Adams directed the attention of the members to a novel proceeding which he had successfully employed at the London Hospital in the reduction of a dislocation backwards of the first phalanx of the thumb. In this case much extension in the ordinary manner had been employed, but without relief. The method consisted in drawing backwards, or extending, as far as possible, the thumb, so as to incline the back of the first and second phalanx on the back of the metacarpal bone; by this the proximal end of the first bone was more closely approximated to the distal end of the metacarpal bone. The thumb was then gradually brought forwards over the end of the metacarpal bone, at the same time that the end of the first phalanx was firmly held in its position. By this means the thumb itself becomes converted into a considerable lever, the fulcrum of which is the proximal end of the first phalanx: the reduction was accomplished with great ease.—*Ibid.*

---

*A Case of Subclavio-axillary Aneurism, successfully treated by Operation.* By F. C. Skey, Esq., F. R. S., Assistant Surgeon to St. Bartholomew's Hospital.—The aneurismal tumor in the case here related was of small size, and was situated at about an inch from the outer border of the left scalenus muscle. It had existed about two months, when the patient put himself under the care of the author; and, as it was rapidly advancing, the operation was immediately determined on. The mode of performing the operation is thus described:—"I commenced an arched incision about three inches above the clavicle, close to the outer border of the sterno-mastoid muscle, and carried a little outwards, curving it in towards the clavicular origin of the muscle, which I exposed to somewhat more than one-half of its length. This flap, convex towards the acromion, I reflected with the platysma muscle. A little careful dissection with a blunt silver knife exposed the lower belly of the omo-hyoideus, and a portion of the sac, through the walls of which the pulsations of the artery were visible. On the inner side the external border of the scalenus was also exposed; and by tearing away the cellular tissue in this space by means of a blunt hook and a silver knife, the transversalis colli and supra-scapular arteries were brought into view, arising from the thyroid axis within the scalenus muscle, and proceeding outwards across the bottom of the wound to their destination. About the transversalis colli was felt the subclavian artery, and above it the lower branches of the axillary plexus of nerves. Having slightly detached it from the rib, I had no difficulty in passing around it an armed needle, at a quarter of an inch on the outer side of the scalenus."

The progress of the case was tolerable favourable until the 17th day after the operation, when appearances manifested themselves in the left leg and thigh which the author attributed to phlebitis. The treatment of this symptom consisted in excoriating the surface over the affected vessels with water nearly boiling, and anointing the excoriated surface with Ung. Hydrarg. mixed with Opium. The ligature separated on the 47th day after the operation.

The author declares his decided preference to the mode of incision adopted in this case over the more usual one along the line of the clavicle, on account of the greater facility of approaching the vessel to be tied, as well as the greater probability of escaping troublesome if not dangerous hæmorrhage.

The paper concludes with observations on the severe constitutional symptoms which frequently follow the ligature of large vessels, and which, in the case now related, were particularly urgent.

*Case of Aneurism of the Right Subclavian Artery, in which a Ligature was successfully applied.* By JOHN P. HALTON, Esq., Surgeon to the Liverpool Infirmary. [Communicated by Sir B. C. Brodie, Bart.]—The patient was a warehouseman, æt. thirty-five, of robust frame, and was admitted into the infirmary in December last, with a strongly pulsating aneurismal tumor immediately below the clavicle, raising that bone considerably from its natural position. The disease had its origin in an accident which occurred three months previously to his admission, when having fallen from a pile of cotton, his fall was suddenly arrested by a hook, which suspended him by his arm. Notwithstanding the appearance of the swelling three weeks after the accident, and the great suffering which attended its rapid increase, he continued his laborious employment until the 24th of December, and did not present himself at the infirmary until the 30th.

Between that time and the 8th of January, when the operation was performed, a rapid increase took place in the size of the tumor. Unlike the author of the paper briefly alluded to above, Mr. Halton prefers the mode of making the first incision which was first recommended by the late Mr. Ramsden, namely, that in the line of the clavicle, from a belief that thereby hæmorrhage is more likely to be avoided. "The integuments were drawn down a little over the clavicle, and, with the platysma myoides, divided by a scalpel upon the upper edge of the bone to the extent of about three inches: the incision commencing nearest the shoulder, and terminating just beyond the sternom-astoid muscle. The integuments above the incision, and on the outer edge of the sterno-mastoid muscle being pinched up, were next separated by one sweep of a sharp pointed bistoury, cutting from within outwards from about the middle of the first incision in a line upwards and backwards, for two inches and a half, due regard being paid to the external jugular vein." By means of the freedom afforded by the external incision just described, the author was enabled to complete the remaining stages of the operation without difficulty, and a double ligature was passed around the artery at a depth of two inches below the clavicle. The hand, arm, and shoulder were enveloped in a thick layer of warm carded cotton. The result of the case was most favourable: the ligature came away on the twelfth day, the incisions healed rapidly, his recovery was uninterrupted by any bad symptom up to the 27th of Feb., on which day the aneurismal swelling had almost disappeared, and pulsation was distinct in the radial artery. After this the swelling became again large and painful, though without pulsation, and matter formed in the situation of the sac. This being evacuated by trocar, to the amount of twelve ounces, was found to consist of very offensive pus, mixed with putrid blood. On the 3d of April the patient was entirely well.—*Transactions of Royal Medical and Chirurgical Society, in Lond. Med. Gaz.*

---

*Pathological and Surgical Observations on the Diseases of the Ear.* By JOSEPH TOYNBEE, Esq. (Presented by Dr. Richard Bright.)—The present paper is the first of a series which the author hopes to lay before the Society on the same subject, and contains the details of forty-one dissections of the internal ear in patients who have died in hospitals and infirmaries of various diseases, and of whose faculty of hearing (as to the greater number, at least,) the author was uninformed. The following is a concise view of the state of the cavity of the tympanum in these cases:—

| | |
|---|---|
| 1. In a healthy state . . . . | 10 |
| 2. With simple thickening of the investing membrane . . . . | 6 |
| 3. With bands of adhesion passing from various parts of the cavity of the tympanum, most frequently connecting the straps to its circumference . . . . . . . | 4 |
| 4. With slight thickening of the investing membrane, accompanied by the existence of adhesive bands . . . . . . | 13 |
| 5. With considerable thickening of the investing membrane and bands of adhesion . . . . . . . | 5 |
| 6. With suppuration of the cavity of the tympanum . . . . . | 1 |
| 7. With anchylosis of the base of the stapes to the circumference of the fenestra ovalis . . . . . | 2 |
| | 41 |

"The large proportion of specimens which are undoubtedly in a diseased state," says the author, "is very surprising; but it may be less so when I state that many persons whom I have examined, and who have considered that they hear perfectly well, cannot distinguish the ticking of my watch at the distance of two feet and a half, and, in some cases, at four or five inches only, though the same watch can be heard by a healthy ear seven or eight feet from the head. I am, therefore, disposed to believe that the function of the ear is impaired much more frequently than is generally supposed."—*Med. Gaz.*

